# The Association between Nutritional Status and In-Hospital Mortality of COVID-19 in Critically-Ill Patients in the ICU

**DOI:** 10.3390/nu13103302

**Published:** 2021-09-22

**Authors:** Michał Czapla, Raúl Juárez-Vela, Vicente Gea-Caballero, Stanisław Zieliński, Marzena Zielińska

**Affiliations:** 1 Department of Public Health, Faculty of Health Sciences, Wroclaw Medical University, 51-618 Wroclaw, Poland; michal.czapla@umed.wroc.pl; 2 Institute of Heart Diseases, University Hospital, 50-566 Wroclaw, Poland; 3 Biomedical Research Centre of La Rioja (CIBIR), Research Group GRUPAC, Research Unit on Health System Sustainability (GISSOS), University of La Rioja, 26004 Logroño, Spain; 4 Faculty of Health Sciences, International University of Valencia, 46002 Valencia, Spain; vicenteantonio.gea@campusviu.es; 5 Department and Clinic of Anaesthesiology and Intensive Therapy, Faculty of Medicine, Wroclaw Medical University, 50-556 Wrocław, Poland; stanislaw.zielinski@umed.wroc.pl (S.Z.); marzena.zielinska@umed.wroc.pl (M.Z.); 6 Department of Anaesthesiology and Intensive Care, University Hospital, 50-556 Wrocław, Poland; 7 Department of Paediatric Anaesthesiology and Intensive Care, University Hospital, 50-556 Wrocław, Poland

**Keywords:** COVID-19, malnutrition, SARS-CoV-2, nutritional status, intensive care unit

## Abstract

Background: Coronavirus disease 2019 (COVID-19) has become one of the leading causes of death worldwide. The impact of poor nutritional status on increased mortality and prolonged ICU (intensive care unit) stay in critically ill patients is well-documented. This study aims to assess how nutritional status and BMI (body mass index) affected in-hospital mortality in critically ill COVID-19 patients Methods: We conducted a retrospective study and analysed medical records of 286 COVID-19 patients admitted to the intensive care unit of the University Clinical Hospital in Wroclaw (Poland). Results: A total of 286 patients were analysed. In the sample group, 8% of patients who died had a BMI within the normal range, 46% were overweight, and 46% were obese. There was a statistically significantly higher death rate in men (73%) and those with BMIs between 25.0–29.9 (*p* = 0.011). Nonsurvivors had a statistically significantly higher HF (Heart Failure) rate (*p* = 0.037) and HT (hypertension) rate (*p* < 0.001). Furthermore, nonsurvivors were statistically significantly older (*p* < 0.001). The risk of death was higher in overweight patients (HR = 2.13; *p* = 0.038). Mortality was influenced by higher scores in parameters such as age (HR = 1.03; *p* = 0.001), NRS2002 (nutritional risk score, HR = 1.18; *p* = 0.019), PCT (procalcitonin, HR = 1.10; *p* < 0.001) and potassium level (HR = 1.40; *p* = 0.023). Conclusions: Being overweight in critically ill COVID-19 patients requiring invasive mechanical ventilation increases their risk of death significantly. Additional factors indicating a higher risk of death include the patient’s age, high PCT, potassium levels, and NRS ≥ 3 measured at the time of admission to the ICU.

## 1. Introduction

On 30 August 2021, 216 million people were infected, and 4.5 million had died of severe coronavirus disease 2019 (COVID-19) [1]. The impact of poor nutritional status on increased mortality and prolonged ICU (intensive care unit) stays in critically ill patients is well known and well-documented [2]. Old age, male sex, comorbidities, being overweight, obesity and malnutrition are some of the known risk factors for severe COVID-19 cases [3]. Moreover, COVID-19 infection lasting several days or even weeks prior to ICU admission enhances patient malnutrition, which in turn leads to increased pathogenicity of the infecting agent and disease progression [4,5]. Furthermore, published studies show a risk of malnutrition in COVID-19 patients. The incidence of malnutrition in patients hospitalised for COVID-19 is 50% [6,7]. Due to malnutrition observed in mechanically ventilated patients even months after discharge from the ICU, both the examination of the nutritional status of critically ill patients at the beginning of hospitalisation and early initiation of nutritional treatment are of great importance [8]. Being overweight and obesity are also factors that worsen prognosis. Individuals coping with these conditions have a higher risk of CVD (cardiovascular disease) and DM (diabetes mellitus). In addition, overweight and obese individuals may experience respiratory complications due to increased ventilatory demand, increased work during breathing, respiratory muscle insufficiency and decreased respiratory compliance [9].

Patients who require treatment in the ICU should be assessed for nutritional status [10]. According to the criteria for diagnosing malnutrition set out by the Global Leadership Initiative on Malnutrition (GLIM), every patient at increased risk for malnutrition should be screened. GLIM indicates that tools such as NRS 2002 (Nutritional Risk Score), SGA (Subjective Global Assessment), MUST (Malnutrition Universal Screening Tool), or MNA (Mini Nutritional Assessment) can be used for screening. In Poland, every person admitted to a hospital ward should undergo a screening test performed using NRS 2002 or SGA (the test does not apply to patients in a hospital Emergency Department; ED) [11,12]. According to ESPEN (European Society for Clinical Nutrition and Metabolism) experts, at-risk patients with more severe COVID-19 should be screened with NRS 2002 if hospitalised [13].

This study aims to assess how nutritional status and BMI (body mass index) affected in-hospital mortality in critically ill COVID-19 patients.

## 2. Materials and Methods

### 2.1. Study Design and Setting

A retrospective study was performed based on an analysis of medical records of active COVID-19 patients (ICD10: U07) admitted to the ICU of the University Clinical Hospital in Wroclaw (Poland) between September 2020 and June 2021. The study followed the STROBE (Strengthening the Reporting of Observational Studies in Epidemiology) guidelines.

### 2.2. Study Population

All the patients who met the following inclusion criteria were analysed: primary diagnosis of COVID-19 (confirmed by RT-PCR), age ≥ 18 years, mechanical ventilation (invasive ventilation), hospitalisation in the ICU.

A final group of 286 patients’ medical records was analysed. The analysis included data (collected at the time admission) concerning patients’ age, sex and BMI; test results such as total cholesterol (TC), triglycerides (TGs), albumins, lymphocytes, potassium, sodium, C-reactive protein (CRP), procalcitonin (PCT); data concerning medical history and comorbidities, as well as assessment of the patients’ nutritional status using NRS 2002.

### 2.3. Nutritional Screening

The NRS 2002 is one of the screening tools recommended by GLIM for risk assessment of nutritional status. The scale consists of two parts.

1. Impaired nutritional status, in which weight loss and BMI are assessed. The same applies to the percentage of food intake compared to its requirements within the last week. The rating scale is 0–3 points, where 0 is lack of deterioration of health status, and 3 is severe deterioration of health status.
2. Severity of disease (an increase in requirements), in which, depending on the disease, patients may receive 0–3 points, where 0 is normal nutritional requirements and 3 is high disease severity (e.g., head injury, bone marrow transplant). Moreover, if patients are over 70, they get an additional point. Thus, patients can score 0–7 points. Nutritional therapy is indicated in patients with NRS ≥ 3 [14]. The WHO criteria were used for classifying patients as underweight (BMI < 18.5 kg/m^2^), with normal weight (BMI: 18.5–24.9 kg/m^2^), pre-obese (BMI: 25–29.9 kg/m^2^) and obese (BMI ≥ 30 kg/m^2^).

A physician measured the NRS 2002 and BMI at the time of admission to the ICU.

### 2.4. Statistical Analysis

Statistical analysis was performed using Statistica 13.1 software (TIBCO, Inc., Palo Alto, CA, USA). First, arithmetic means and standard deviations were calculated for measurable variables. Next, quantitative variables were examined using the Shapiro-Wilk test to determine the distribution type. Then, intergroup comparisons were made using the t-test or the Mann-Whitney U test (if assumptions were met). Finally, the comparison of results of more than two groups was performed using one-way analysis of variance (ANOVA) or the Kruskal-Wallis test (if assumptions were met).

Survival analysis was carried out using the Kaplan-Meier method and refers to ICU mortality. The log-rank test was used for comparing patient survival against selected clinical variables. The Cox proportional hazards model was used for assessing the influence of qualitative or quantitative variables on patient survival. The analysis included both categorical variables and continuous variables. The categorical variables included sex, BMI (18.5–24.9, <18.5, 25.0–29.9, ≥30), NRS (<3 vs. ≥3), HF (heart failure, yes/no), HT (hypertension, yes/no), DM (diabetes mellitus, yes/no), CVD (cardiovascular disease, yes/no), CKD (chronic kidney disease, yes/no), CRD (chronic respiratory disease, yes/no), TGs (triglycerides, <135, 135–200, >200), TC (total cholesterol, <40, >40). The continuous variables included age, BMI [kg/m^2^], height [m], body weight [kg], TGs [mg/dL], TC [mg/dL], CRP [mg/L], albumins [g/dL], lymphocytes [%], PCT [ng/mL], potassium [mmol/l], sodium [mmol/L].

Variables such as BMI, TC, or NRS were analysed as continuous and categorical variables in the univariate model. Variables were included in the multivariate model in accordance with the adopted criteria. Those criteria included the outcome of *p* < 0.30 in a univariate model, a lack of correlation of variables, and clinical recommendations. Multivariate analysis was performed using backwards elimination to stay in the model. For the final multivariate model, the variables were selected according to the better fit of the model based on the assessment of the goodness of fit (AIC). The results were considered statistically significant at *p* < 0.05.

## 3. Results

### 3.1. Characteristics of the Group

The profile of the whole group with a comparison of the analysed characteristics of the survivors and nonsurvivors is shown in Table 1. A total of 286 patients were analysed. Due to a lack of data for some parameters, those numbers are smaller and are provided for each variable. There was a statistically significantly higher death rate in men (73%, *n* = 142) and those with BMI between 25.0–29.9 (46% vs. 26%; *p* = 0.011). Nonsurvivors had a statistically significantly higher HF rate (9% vs. 2%; *p* = 0.037) and HT rate (55% vs. 24%; *p* < 0.001). Furthermore, nonsurvivors were statistically significantly older (x = 63.6 vs. x = 53.8 years; *p* < 0,001), taller (x = 175.9 vs. x = 172.1 cm; *p* = 0.008). Considering laboratory test parameters, PCT levels were statistically significantly higher in nonsurvivors. TC levels were statistically significantly lower in nonsurvivors (Table 1).

### 3.2. Subgroup Analysis According to BMI

A comparison of the assessed variables between groups according to BMI is shown in Table 2 and Table 3. Men showed a statistically significantly higher percentage in the BMI ranges of 18.5–24.9 kg/m^2^, 25.0–29.9 and above 30 kg/m^2^, compared to women. The highest percentage of deaths was observed in patients with BMI between 25.0 and 29.9 kg/m^2^ (Table 2). The highest (statistically significant) CRP levels were observed in the group of patients with BMI between 18.5 and 24.9 kg/m^2^.

### 3.3. Subgroup Analysis According to NRS

Comparisons of the assessed parameters between groups according to NRS scores are shown in Table 4 and Table 5. Based on the NRS score, two groups were distinguished: NRS <3 and ≥3. There were no statistically significant differences between the groups.

### 3.4. Survival Analysis

**Figure 1:**
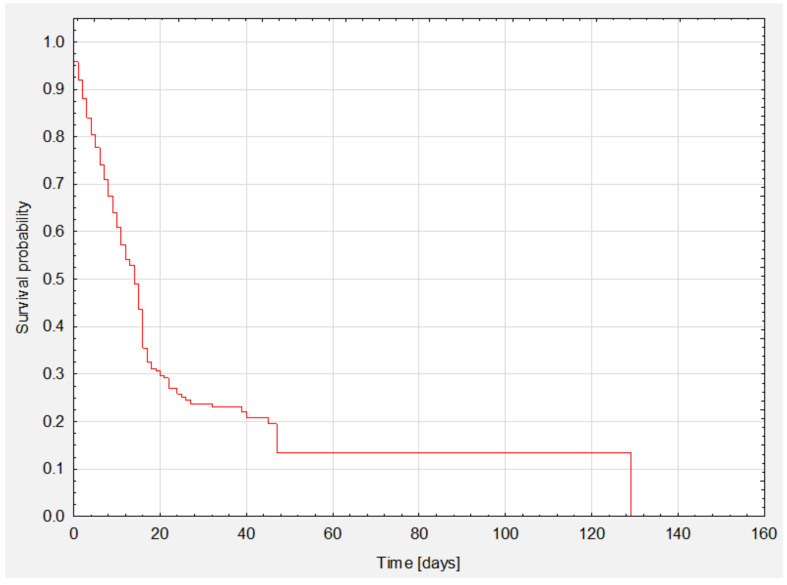
Analysis of survival of the whole group.

Survival analysis is shown in Kaplan-Meier survival curves (Figure 1). The median survival was 14 days (Table 6). Total survival was 32.2% (*n* = 92).

### 3.5. Survival Analysis—Group Comparisons

**Figure 2:**
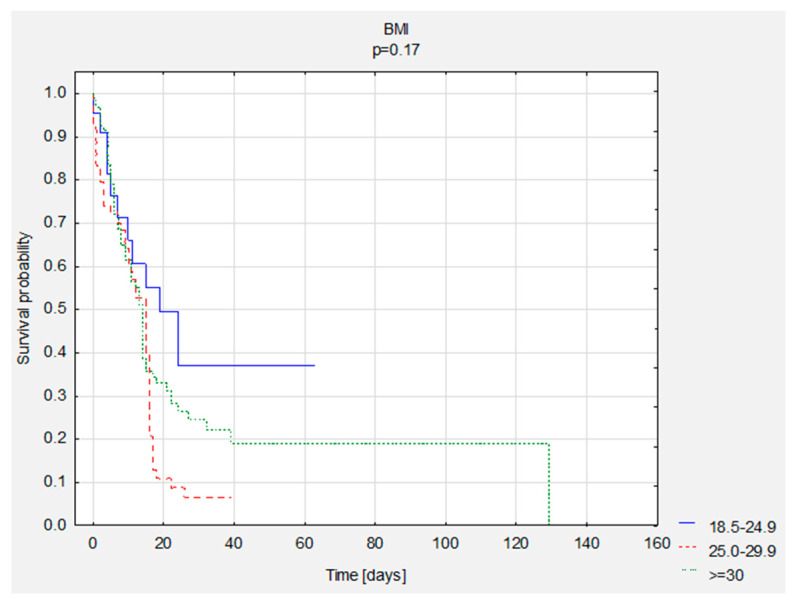
Comparison of survival curves according to BMI scores.

**Figure 3:**
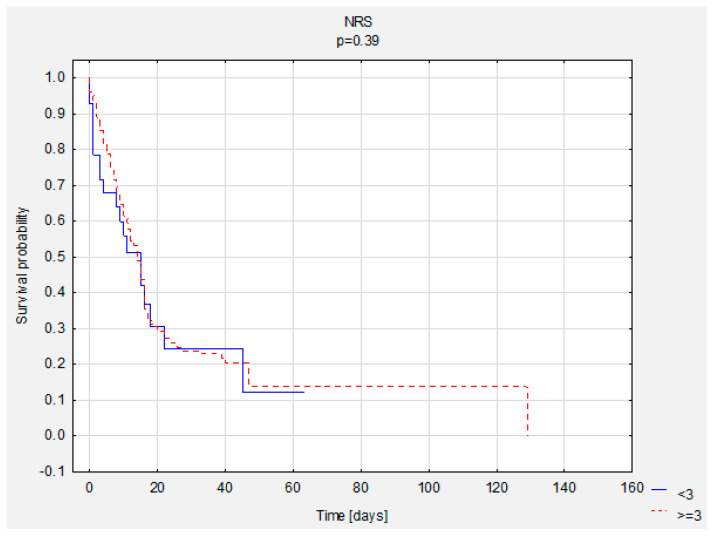
Comparison of survival curves according to NRS scores.

A comparison of survival curves was performed according to BMI and NRS. There were no statistically significant differences (Table 7, Figure 2 and Figure 3).

Assessment of the influence of selected variables on mortality is shown in Table 8 (Cox proportional hazards regression). It was observed that the risk of death increased in the group with BMI in the range of 25.0–29.9 (HR = 2.18; *p* = 0.010). Taking the quantitative variables into account, the risk of death was lower in patients with higher levels of TC (HR = 0.996; *p* = 0.034) and sodium (HR = 0.97; *p* = 0.033). However, age (HR = 1.03; *p* < 0.001), NRS (HR = 1.18; *p* = 0.019), high potassium (HR = 1.34; *p* = 0.002) and PCT (HR = 1.04; *p* < 0.001) also affected mortality.

Variables were included in the multivariate model in accordance with the adopted criteria. The criteria included the outcome of *p* < 0.30 in a univariate model, a lack of correlation of variables, and clinical recommendations. The following variables were included in the model: BMI (categories), HF, TC (quantitatively), age, NRS (quantitatively), potassium, sodium, CKD, CRP and PCT (Table 9).

The multivariate analysis showed that age (HR = 1.03, *p* ≤ 0.001), potassium (HR = 1.40, *p* = 0.023), PCT (HR = 0.10, *p* < 0.001) and BMI 25.00–29.99 correlated with mortality (HR = 2.13, *p* = 0.038). Table 9 shows statistically significant variables.

## 4. Discussion

The nutritional status of COVID-19 patients is undoubtedly related to complications, and increased risk of death during in-patient treatment. The present study showed that patients with COVID-19 who died in the ICU were statistically significantly more likely to have comorbidities such as HF (*p* = 0.037) or HT (*p* = 0029). Nearly 68% of patients did not survive to discharge. Men died statistically significantly more often (*p* = 0.005). Other authors report between 20 and 62% of deaths during hospitalisation in the ICU and in the case of mechanically (either noninvasively or invasively) ventilated patients, from 50% to as much as 97% [15,16,17].

In the study group, the risk of death more than doubled (HR = 2.18) in patients who were overweight. The reasons for this may be that overweight patient are often not aware of their health condition because they do not have symptoms (e.g., metabolic disorders such as hypertension, insulin resistance, dyslipidemia or prediabetes, which frequently occur in overweight patients); therefore, they are less likely to undergo health examinations to diagnose their diseases, and comorbidities can lead to an increasingly severe course of, and consequent death from, COVID-19. It is widely known that both being overweight and obesity are risk factors for developing many comorbidities (including hypertension, CVD, DM2, obstructive sleep apnea) considered as risk factors for severe complications of COVID-19 [18,19,20]. In addition, being overweight and obesity alone may cause, e.g., chronic inflammation, which may lead to lowered immunity and lung function impairment. Jingzhou et.al. showed that being overweight was significantly associated with COVID-19 mortality at a global level [21].

Excessive body fat impedes respiratory gas exchange. Subcutaneous adipose tissue in the frontal part of the chest and at its sides increases its resistance during respiration, and, as a consequence, the patient might require higher positive pressure during mechanical ventilation [22]. It is also worth emphasising that abnormal body weight could be a problem during patient extubation. In such cases, especially in patients with obesity, there is a higher probability of upper airway obstruction and re-intubation [23]. According to Kompaniyets et al., in the United States from March to December 2020, among 34,899 patients hospitalised in the ICU for COVID-19, almost 28% were overweight and 50% were obese. The risk of death increased with increasing BMI [24]. In Europe (England), Gao et al. revealed in their prospective study that among 1601 patients in the ICU, 31% were overweight, and 50% were obese [25]. The results of the current study are similar. Among all inpatients, more than 40% were overweight, and nearly 50% were obese. Similar results were reported in France, where being overweight and obesity concerned 41.4% and 43.4% of ICU in-patients, respectively. In the cited study, the death rate was lower and amounted to 18.5%. Interestingly, multifactorial analysis indicated a paradoxical relationship between the category of BMI and mortality. Patients whose BMI was ≤29 kg/m^2^ (OR = 3.64) and those whose BMI was >39 kg/m^2^ (OR = 10.45) were more at risk of death compared to those with a BMI of 29–39 kg/m^2^ [26]. Researchers point out that the risk of a severe COVID-19 disease course and invasive ventilation in the ICU is higher in patients being overweight or with obesity [27,28]. On the other hand, they also demonstrated that obesity is not a predictor of a higher risk of death in the ICU. A study by Zhaozhong et al. confirmed that there were more patients with obesity admitted to the ICU compared to those with a BMI < 30 kg/m^2^ but, at the same time, obesity did not affect survival in these hospital units [29]. In another study, severe obesity classified as a BMI > 35 kg/m^2^, and male sex, were independently related to the need for patient intubation and death [30]. Some studies, in this case, refer to the so-called “obesity paradox”. Ironically, in patients with CVD, despite increased health risks associated with obesity, treatment outcomes were often better than in slimmer patients [31]. This can be observed, for example, in patients with HF. One of the possible explanations for this phenomenon is that the disease frequently develops in patients with obesity at a younger age, and intensive therapy, which might result in reduced mortality in this group, is provided earlier. In our study there was no significant difference regarding the age of obese and nonobese patients, but in some studies people with obesity were up to 10 years younger than individuals with normal body mass [32]. In these patients, their adipose tissue could serve as a nutrient when metabolism declines [33,34]. BMI itself is not a good indicator of obesity because it does not consider exact body composition, i.e., amount of muscle, fat distribution or water retention. However, due to its ease of use and availability, it is an integral part of the evaluation of patients with other diseases as well [35]. The index does not distinguish well between obesity phenotypes; thus, the same patient with a high BMI may be an individual with an athletic physique or sarcopenic obesity. In a recently published literature review, Dalamaga et al. confirmed that obesity and increased visceral fat were significant risk factors for poor outcomes related to COVID-19. Even though the presented study did not find any relation between obesity and the risk of death, its presence should still be considered a potential risk factor for severe complications and death.

In this study, univariate analysis revealed that the risk of death increased with the risk of malnutrition according to NRS2002 (HR = 1.18). Osuna-Padilla et al. showed that nutritional risk in mechanically ventilated ICU patients was related to an increased risk of death (OR = 2.4, and was found in 66% of patients [36]. A similar result was reported by Zhang et al., where nutritional risk was found in more than 60% of hospitalised patients. The mortality rate was statistically significantly higher in that group. Moreover, those patients suffered from more comorbidities [37]. In both studies, the nutritional risk was analysed using the NUTRIC score. In this study, the risk of malnutrition occurred in 90% of patients and the screening assessment of nutritional status was performed using the validated NRS2002 questionnaire. Studies by other authors show that it is a good tool to assess the nutritional status of COVID-19 patients [38]. Retrospective and review studies show a high percentage of patients who are not only critically ill are also affected by malnutrition. This number is as high as 70%. These studies also show that malnutrition risk is strongly associated with mortality [39,40,41]. Malnutrition results in decreased body weight, muscle weakness, impaired immunity, decreased protein levels and oxygen utilisation [42], which affects the course of the disease.

In the multivariate analysis, in addition to being overweight, increases in plasma potassium levels (HR = 1.40) and procalcitonin levels (HR = 1.10) were associated with higher mortality risk. Abnormal plasma potassium levels may be one of the symptoms of acid-base imbalance that occurs in patients with acute respiratory failure. It may also cause cardiac arrhythmia, bradyarrhythmia, complete heart block and circulatory arrest [43,44]. In a study concerning critically ill COVID-19 patients, Shengcong et al. found a significant increase in mortality rate in patients with potassium levels ≥5.0 mmol/L [45]. On the other hand, according to Perez, hypokalemia is associated with longer hospital and ICU stay but does not affect mortality [46]. Research shows that an increase in procalcitonin levels is one of the indicators of disease severity in COVID-19 patients, and is a risk factor of mortality [47,48]. The results of this study seem to be consistent with those published so far and indicate an association between the value of this inflammatory marker and the increased risk of in-hospital mortality (in multivariate analysis: HR = 1.10; *p* < 0.001) [49,50]. In the study by Fenk et al., the risk of death increased up to fivefold (OR = 5.65; *p* < 0.001) in patients with high procalcitonin levels, indicating that this marker may be useful to reflect the degree of lung involvement during SARS-CoV-2 infection [51]. Leoni et al., who studied 242 COVID-19 patients hospitalised in the ICU, also found that predictors of increased mortality rate in the ICU include, among others, age, obesity and higher procalcitonin levels (HR = 1.03, *p* = 0.04). These predictors were independently associated with 28-day mortality [52].

### Study Limitations

This study has some limitations. As it involved critically ill and mechanically ventilated (using invasive methods) patients, complete data concerning their medical history and medications could not always be obtained due to the serious nature of the situation.

Besides, we obtained statistical significance according to our classification, although other classifications could have been done. In our study, the data with no statistical significance must be taken with caution, and cannot be excluded.

The inference analysis should be interpreted with the variables we selected, and considering the whole study. We also do not have information about the time between the first symptoms of COVID-19 and hospital admission to the ICU. In some cases, NRS and BMI scores were not included in medical records. Regarding to the high proportion (90%) of individuals with a NRS ≥ 3, this can interfere with the results. Moreover, body composition analysis was not conducted in patients, and BMI scores may not be a reliable indicator for assessing overweight and obesity. Patients did not have their waist-to-hip ratio (WHR) measured, and data concerning central obesity based on waist circumference was not reported either. Finally, the long-term survival of COVID-19 patients could not be assessed due to restrictions on access to personal data because of the anonymity of medical records.

## 5. Conclusions

Being overweight in critically ill COVID-19 patients requiring invasive mechanical ventilation increases their risk of death significantly. Additional factors indicating a higher risk of death include the patient’s age, high PCT and potassium levels measured at the time of admission to the ICU. Even though the presented study did not find any association between obesity and the risk of death, obesity should still be considered a potential risk factor for severe complications and death. The lack of confirmation of this association in this study should not be interpreted as providing a potential protective effect. The risk of malnutrition at the time of ICU admission also increases the risk of in-hospital death. Undoubtedly, studies concerning the nutritional status of COVID-19 patients hospitalised in the ICU need further investigation.

## Figures and Tables

**Table 1:** Characteristics of the group with a comparison of survivors and nonsurvivors.

Variables		Total (*n* = 286)		Death				*p*-Value *
				No (*n* = 92)		Yes (*n* = 194)		
		*n*	%	*n*	%	*N*	%	
Sex (*n* = 286)	M	194	67.8	52	56.5	142	73.2	0.005
BMI (*n* = 194)	<18.5	-	-	-	-	-	-	0.011
	18.5–24.9	22	11.3	11	19.3	11	8.03	
	25.0–29.9	78	40.2	15	26.3	63	45.9	
	≥30	94	48.5	31	54.4	63	45.9	
NRS (*n* = 286)	<3	28	9.8	9	9.8	19	9.8	0.991
	≥3	258	90.2	83	90.2	175	90.2	
HF (*n* = 286)	Yes	19	6.64	2	2.2	17	8.8	0.037
HT (*n* = 286)	Yes	145	50.7	38	41.3	107	55.2	0.029
DM (*n* = 286)	Yes	92	32.2	25	27.2	67	34.5	0.214
CVD (*n* = 286)	Yes	99	34.6	30	32.6	69	35.6	0.622
CRD (*n* = 286)	Yes	24	9.4	6	6.52	18	9.3	0.433
CKD (*n* = 286)	Yes	8	2.8	1	1.1	7	3.6	0.232
TC (*n* = 232)	>190	49	21.1	20	24.4	29	19.3	0.900
TGs (*n* = 251)	>150	183	72.9	58	73.4	125	72.7	0.372
**Variables**		**x**	**SD**	**x**	**SD**	**x**	**SD**	***p*** -value **
Age (*n* = 286)		60.5	13.2	53.8	13.5	63.6	11.8	<0.001
ICU length stay (*n* = 286)		14.2	14.4	20.2	16.0	11.0	12.6	<0.001
NRS (*n* = 286)		3.3	1.1	3.1	1.1	3.4	1.1	0.061
BMI (*n* = 194)		31.0	5.7	31.6	6.4	30.7	5.4	0.291
TGs [mg/dL] (*n* = 251)		250.3	148.3	236.7	160.5	256.5	142.5	0.333
TC [mg/dL] (*n* = 232)		144.2	50.7	155.8	47.9	137.9	51.2	0.010
Albumins [g/dL] (*n* = 276)		2.9	0.4	2.9	0.4	2.9	0.4	0.652
Lymphocytes [%] (*n* = 271)		9.3	10.4	9.4	7.7	9.3	11.5	0.981
Potassium [mmol/L] (*n* = 280)		4.4	0.8	4.3	0.7	4.5	0.9	0.092
Sodium [mmol/L] (*n* = 280)		139.6	5.4	140.2	4.2	139.2	5.8	0.141
CRP [mg/L] (*n* = 281)		140.1	100.2	132.7	87.1	143.5	105.7	0.400
PCT [ng/mL] (*n* = 280)		2.1	8.7	0.5	0.8	2.9	10.4	0.030

Abbreviations: *n*, number of participants; x mean; SD, standard deviation; M, males; *p*, level of significance; BMI, body mass index; NRS, nutritional risk screening; HF, heart failure; HT, arterial hypertension; DM, diabetes mellitus; CVD, cardiovascular disease; CRD, chronic respiratory disease; CKD, chronic kidney disease; TGs, triglycerides; TC, total cholesterol; CRP, C-reactive protein; PCT, procalcitonin; * χ^2^ test; ** *t*-test.

**Table 2:** Comparison of assessed parameters (qualitative variables) and BMI range (WHO criteria) values.

Variables		BMI						*p*-Value *
		18.5–24.9 *n* = 22		25.0–29.9 *n* = 78		≥30 *n* = 94		
		*n*	%	*n*	%	*n*	%	
Sex	M	21	95.5	65	83.3	53	56.4	<0.001
NRS	<3	2	9.09	8	10.26	4	4.26	0.300
	≥3	20	90.91	70	89.74	90	95.74	
Death	Yes	11	50.00	63	80.77	63	67.02	0.011
HF	Yes	1	4.55	6	7.69	6	6.38	0.861
HT	Yes	9	40.91	41	52.56	55	58.51	0.311
DM	Yes	5	22.73	28	35.90	35	37.23	0.433
CVD	Yes	6	27.27	27	34.62	28	29.79	0.722
CRD	Yes	0	0.00	9	11.54	10	10.64	0.261
CKD	Yes	1	4.55	2	2.56	2	2.13	0.811
TC	>190	4	21.05	10	16.13	16	20.25	0.792
TGs	>150	11	57.89	53	74.65	67	81.71	0.081

Abbreviations: *n*, number of participants; M, males; *p*, level of significance; BMI, body mass index; NRS, nutritional risk screening; HF, heart failure; HT, arterial hypertension; DM, diabetes mellitus; CVD, cardiovascular disease; CRD, chronic respiratory disease; CKD, chronic kidney disease; TGs, triglycerides; TC, total cholesterol; CRP, C-reactive protein; PCT, procalcitonin; * χ^2^ test.

**Table 3:** Comparison of assessed parameters (quantitative variables) and BMI range (WHO criteria) values.

Variables	BMI									*p*-Value **
	18.5–24.9 *n* = 22			25.0–29.9 *n* = 78			≥30 *n* = 94			
	*n*	x	SD	*n*	x	SD	*n*	x	SD	
Age	22	56.6	17.2	78	62.8	10.0	94	60.2	12.2	0.081
NRS	22	3.4	1.4	78	3.4	1.2	94	3.4	1.0	1.001
TGs [mg/dL]	19	215.8	160.8	71	242.9	123.7	82	259.4	128.7	0.392
TC [mg/dL]	19	143.1	46.1	62	131.2	50.7	79	149.7	48.8	0.091
Albumins [g/dL]	21	3.0	0.4	76	2.9	0.5	91	3.0	0.4	0.202
Lymphocytes [%]	18	6.0	3.7	76	10.2	14.3	90	9.3	6.7	0.322
Potassium [mmol/L]	21	4.4	0.9	77	4.5	0.9	92	4.3	0.6	0.281
Sodium [mmol/L]	21	138.8	4.0	77	140.6	5.4	92	139.4	5.8	0.242
CRP [mg/L]	21	183.5	115.9	77	122.3	100.8	92	133.8	89.5	0.040
PCT [ng/mL]	21	2.5	8.6	76	1.7	4.7	92	1.9	9.3	0.913

Abbreviations: *n*, number of participants; x, mean; SD, standard deviation; *p*, level of significance; BMI, body mass index; TGs, triglycerides; TC, total cholesterol; CRP, C-reactive protein; PCT, procalcitonin; ** *t*-test.

**Table 4:** Comparison of assessed parameters (qualitative variables) and NRS scores.

Variables		NRS				*p*-Value *
		<3 *n* = 28		≥3 *n* = 258		
		*n*	%	*n*	%	
Sex	M	18	64.39	176	68.22	0.671
BMI	<18.5	2	14.29	20	11.11	0.301
	18.5–24.9	8	57.14	70	38.89	
	25.0–29.9	4	28.57	90	50.00	
Death	Yes	19	67.86	175	67.83	0.994
HF	Yes	1	3.57	18	6.98	0.493
HT	Yes	11	39.29	134	51.94	0.202
DM	Yes	9	32.14	83	32.17	0.992
CVD	Yes	13	46.43	86	33.33	0.171
CRD	Yes	4	14.29	20	7.75	0.244
CKD	Yes	0	0.00	8	3.10	0.343
TC	>190	7	30.43	42	20.10	0.253
TGs	>150	17	68.00	166	73.45	0.561

Abbreviations: *n*, number of participants; M, males; *p*, level of significance; BMI, body mass index; NRS, nutritional risk screening; HF, heart failure; HT, arterial hypertension; DM, diabetes mellitus; CVD, cardiovascular disease; CRD, chronic respiratory disease; CKD, chronic kidney disease; TGs, triglycerides; TC, total cholesterol; CRP, C-reactive protein; PCT, procalcitonin; * χ^2^ test.

**Table 5:** Comparison of assessed parameters (quantitative variables) and NRS scores.

Variables	NRS						*p*-Value **
	<3 *n* = 28			≥3 *n* = 258			
	*n*	x	SD	*n*	x	SD	
Age	28	57.3	13.2	258	60.8	13.2	0.181
BMI	14	29.6	6.7	180	31.1	5.7	0.362
Height [cm]	13	174.5	8.2	180	174.8	9.1	0.901
Body Mass [kg]	13	85.6	13.1	181	94.8	17.7	0.071
TGs [mg/dL]	25	240.1	150.0	226	251.4	148.5	0.722
TC [mg/dL]	23	148.9	61.1	209	143.7	49.6	0.644
Albumins [g/dL]	27	2.8	0.5	249	2.9	0.4	0.071
Lymphocytes [%]	26	8.2	3.9	245	9.5	10.9	0.551
Potassium [mmol/L]	27	4.6	1.1	253	4.4	0.8	0.242
Sodium [mmol/L]	27	141.1	5.4	253	139.4	5.4	0.121
CRP [mg/L]	28	123.5	93.7	253	141.9	100.9	0.364
PCT [ng/mL]	28	3.6	16.0	252	2.0	7.5	0.351

Abbreviations: *n*, number of participants; x, mean; SD, standard deviation; *p*, level of significance; BMI, body mass index; TGs, triglycerides; TC, total cholesterol; CRP, C-reactive protein; PCT, procalcitonin; ** *t*-test.

**Table 6:** Survival time.

		Survival Time [Days]
Percentiles	25 percentiles (lower quartile)	6.0
	50 percentiles (median)	14.3
	75 percentiles (upper quartile)	25.3

**Table 7:** Descriptive statistics for survival time, number of deaths and survival according to BMI and NRS scores.

		Descriptive Statistics				
		Me	x	SD	*n*—Death	*n*—Survivors
BMI	<18.5	-	-	-	-	-
	18.5–24.9	13.0	18.0	17.8	11	11
	25.0–29.9	11.5	10.8	8.2	63	15
	≥30	11.0	14.7	16.6	63	31
NRS	<3	10.0	13.2	14.8	19	9
	≥3	11.0	14.4	14.3	175	83

Abbreviations: *n*, number of participants; Me, median; x, mean; SD, standard deviation; BMI, body mass index; NRS, nutritional risk screening.

**Table 8:** Assessment of the influence of variables on mortality: the Cox proportional hazards regression model, a single model.

		*p*-Value	HR	95% CI HR (Lower)	95% CI HR (Upper)
Sex (*n* = 286)	M	0.451	1.13	0.82	1.56
BMI (*n* = 194)	18.5–24.9	Ref.			
	25.0–29.9	0.010	2.18	1.14	4.16
	≥30	0.662	1.62	0.85	3.07
NRS (*n* = 286)	<3	Ref.			
	≥3	0.661	0.90	0.56	1.44
HF (*n* = 286)	Yes	0.281	1.32	0.79	2.21
HT (*n* = 286)	Yes	0.733	1.05	0.79	1.40
DM (*n* = 286)	No	0.344	1.15	0.86	1.55
CVD (*n* = 286)	Yes	0.941	1.01	0.75	1.36
CRD (*n* = 286)	Yes	0.080	1.55	0.95	2.52
CKD (*n* = 286)	Yes	0.641	1.20	0.56	2.55
TGs (*n* = 251)	>150	0.671	1.08	0.77	1.51
TC (*n* = 232)	>190	0.184	0.76	0.51	1.14
Variables					
Age (*n* = 286)		0.000	1.03	1.02	1.04
NRS (*n* = 286)		0.019	1.18	1.03	1.35
BMI (*n* = 194)		0.522	0.99	0.96	1.02
Height [cm] (*n* = 193)		0.762	1.00	0.98	1.02
Body Mass [kg] (*n* = 194)		0.733	1.00	0.99	1.01
TGs [mg/dL] (*n* = 251)		0.844	1.00	1.00	1.00
TC [mg/dL] (*n* = 232)		0.034	1.00	0.99	1.00
Albumins [g/dL] (*n* = 276)		0.844	1.04	0.74	1.44
Lymphocytes [%] (*n* = 271)		0.811	1.00	0.99	1.02
Potassium [mmol/L] (*n* = 280)		0.002	1.34	1.11	1.61
Sodium [mmol/L] (*n* = 280)		0.033	0.97	0.95	1.00
CRP [mg/L] (*n* = 281)		0.283	1.00	1.00	1.00
PCT [ng/mL] (*n* = 280)		0.000	1.04	1.03	1.05

Abbreviations: *n*, number of participants; M, males; HR, hazard ratio; CI, confidence interval; *p*, level of significance; BMI, body mass index; NRS, nutritional risk screening; HF, heart failure; HT, arterial hypertension; DM, diabetes mellitus; CVD, cardiovascular disease; CRD, chronic respiratory disease; CKD, chronic kidney disease; TGs, triglycerides; TC, total cholesterol; CRP, C-reactive protein; PCT, procalcitonin.

**Table 9:** Assessment of the influence of variables on mortality: the Cox proportional hazards regression model, a multivariate model.

*n* = 153		Beta	Standard Error	Chi-Square	*p*-Value	HR	95% CI HR (Lower)	95% CI HR (Upper)
Age		0.03	0.01	11.2	0.001	1.03	1.01	1.05
Potassium [mmol/L]		0.34	0.15	5.2	0.023	1.40	1.05	1.88
PCT [ng/mL]		0.09	0.02	23.5	<0.001	1.10	1.06	1.14
BMI	25.0–29.9	0.33	0.16	4.3	0.038	2.13	1.03	4.40
	≥30	0.09	0.16	0.3	0.561	1.68	0.81	3.47

Abbreviations: *n*, number of participants; M, males; HR, hazard ratio; CI, confidence interval; *p*, level of significance; BMI, body mass index; TC, total cholesterol, PCT, procalcitonin.

## Data Availability

The data are available by contacting the corresponding author.
